# Conformational Ensembles of an Intrinsically Disordered Protein pKID with and without a KIX Domain in Explicit Solvent Investigated by All-Atom Multicanonical Molecular Dynamics

**DOI:** 10.3390/biom2010104

**Published:** 2012-02-22

**Authors:** Koji Umezawa, Jinzen Ikebe, Mitsunori Takano, Haruki Nakamura, Junichi Higo

**Affiliations:** 1 Graduate School of Advanced Science and Engineering, Waseda University, Okubo 3-4-1, Shinjuku-Ku, Tokyo 169-8555, Japan; Email: k.umezawa@aoni.waseda.jp (K.U.); mtkn@waseda.jp (M.T.); 2 Institute for Protein Research, Osaka University, Suita, Osaka, 565-0871, Japan; Email: jinzen@protein.osaka-u.ac.jp (J.I.); harukin@protein.osaka-u.ac.jp (H.N.)

**Keywords:** IDP, phosphorylated kinase inducible domain, kinase-induced domain interacting domain, coupled folding and binding, free energy landscape, mixed lineage leukemia (MLL)

## Abstract

The phosphorylated kinase-inducible activation domain (pKID) adopts a helix–loop–helix structure upon binding to its partner KIX, although it is unstructured in the unbound state. The N-terminal and C-terminal regions of pKID, which adopt helices in the complex, are called, respectively, α_A_ and α_B_. We performed all-atom multicanonical molecular dynamics simulations of pKID with and without KIX in explicit solvents to generate conformational ensembles. Although the unbound pKID was disordered overall, α_A_ and α_B_ exhibited a nascent helix propensity; the propensity of α_A_ was stronger than that of α_B_, which agrees with experimental results. In the bound state, the free-energy landscape of α_B_ involved two low free-energy fractions: native-like and non-native fractions. This result suggests that α_B_ folds according to the induced-fit mechanism. The α_B_-helix direction was well aligned as in the NMR complex structure, although the α_A_ helix exhibited high flexibility. These results also agree quantitatively with experimental observations. We have detected that the α_B_ helix can bind to another site of KIX, to which another protein MLL also binds with the adopting helix. Consequently, MLL can facilitate pKID binding to the pKID-binding site by blocking the MLL-binding site. This also supports experimentally obtained results.

## 1. Introduction

A traditional view related to protein function, is that a folded three-dimensional structure plays a fundamental role as a scaffold to hold the function. However, this view has been modified by discovery of intrinsically disordered proteins (IDPs), which are proteins (or protein regions) that lack a well-defined three-dimensional structure in the isolated state (*i.e.*, unbound/free state), existing as an ensemble of interconverting conformations. Many IDPs interact with partner molecules, transferring to the folded state (*i.e.*, bound state). The tertiary structures of IDPs in the bound state have been determined experimentally. This remarkable feature is called coupled folding and binding [1], which combines two major subjects––protein folding and molecular recognition––each of which has been studied individually in the protein science field.

Actually, IDPs differ from ordered proteins in several respects: They are found ubiquitously in transcriptional regulators of eukaryote [2] and they frequently undergo posttranscriptional modification [3] such as phosphorylation. Furthermore, some severe diseases are related to IDPs [4]. Consequently, they have been recognized as potential drug targets [5,6]. A biophysically interesting point is that the intrinsic disorder in the unbound state offers advantages over folded proteins [7]. For instance, molecular association is enhanced by the intrinsic disorder [8].

Experiments do not provide sufficient information related to early events in the coupled folding and binding, where the IDP and its partner molecule are separate or weakly interacting with one another. Consequently, theoretical [9] and computational [10,11,12,13] studies might be crucially important to reveal important aspects of their early events. One fascinating mechanism from these studies is a “fly casting” model [9], in which the disordered state allows IDP to capture a distant partner molecule because the disordered polypeptide has a greater interaction radius than a well ordered structure does. After capturing the partner, the disordered polypeptide is reeled to form the native complex. However, Huang *et al.* [12] have implied that the kinetic advantage derived from the greater interaction radius must be countervailed by its slow diffusion. They have suggested a picture in which the kinetic advantage may not be derived from the greater interaction radius but from a lowered free energy barrier. Then the IDP reaches the final bound form through fewer encounter complexes with its partner than an ordered protein does. Consequently, the association mechanism of IDP remains controversial. All of these binding schemes were derived from simplified protein models. We suggest that an all-atom computation can provide useful insight into this experimentally undetectable process.

Most computational studies [11,12,13,14,15] have been conducted using simplified protein models such as a Gō-like coarse-grained model, where one amino-acid residue is expressed usually by one sphere. This model has been used widely to investigate protein folding and molecular recognition because of its low computational cost. A typical Gō-like model postulates that natively formed residue–residue contacts (native interactions) in the native structure are energetically favored, even at a transition state. The other contacts (non-native interactions) likely slow the folding rate. However, the non-native interactions might speed up the folding rate when they help the unfolded polypeptide to collapse, as occurs with competition with chain entropy [16]. If this scheme is correct, then non-native interactions might also positively support or facilitate the IDP–partner association. However, the protein models so far used are too simple to support a realistic discussion of the native and non-native factors. To elucidate these factors, an all-atom protein model is expected to be useful.

The all-atom model involves all the interaction factors. However, it is usually difficult to construct a statistically significant conformational ensemble of protein because the conformational space (potential energy surface) is constructed with a huge number of degrees of freedom and the conformation is frequently trapped in energy local minima during a simulation. Consequently, sampling the ensemble requires unrealistically long computation time by conventional simulations. We have overcome this difficulty using an enhanced sampling method: multicanonical molecular dynamics (McMD) [17,18]. The advantage of McMD is that the energy barriers among the energy local minima are overcome, as explained later. Recently, we developed a more efficient sampling method, trivial trajectory parallelization of multicanonical molecular dynamics (TTP-McMD) [19,20]. Using TTP-McMD, we have conducted an all-atom folding simulation of a 57 amino-acid residue protein [21] and the coupled folding and binding of NRSF and mSin3 [22], in explicit solvent. The NRSF is IDP and folds into the helix when binding to the partner mSin3. The simulation reproduced a conformational ensemble at room temperature, which contained the native-like complex structure in the largest population cluster (*i.e.*, the most thermodynamically stable cluster/the lowest free-energy cluster) as well as non-native complex structures in minor clusters. Therefore, the TTP-McMD is a useful computational technique to examine IDP systems.

The cAMP-response element-binding protein (CREB) induces transcription via an interaction with its co-activator CREB binding protein (CBP). The transcription factor CREB contains a kinase-inducible domain (KID) to bind the kinase-induced domain interacting domain (KIX) of CBP [23,24]. The binding affinity of KID with KIX depends on phosphorylation [25,26]: the affinity increases as Ser133 of KID is phosphorylated. Both KID and the phosphorylated KID (pKID) are IDPs [27], and the tertiary structure of the pKID–KIX complex was determined using NMR at 315 K (PDB ID: 1kdx [27]). The deposited 17 NMR models show that pKID adopts a helix–loop–helix structure on the KIX surface. Therefore the binding of pKID to KIX is cooperated with folding [28]. Sugase *et al.* studied the kinetics of pKID binding with KIX by NMR [29]. This system is suitable for all-atom computations because the pKID sequence deposited in PDB is short (28 residues).

We note some experimental features of this pKID–KIX system. First, the binding affinity of the C-terminal helix of pKID (called α_B_; residues 134–145 in the original PDB file) is one order stronger than that of the N-terminal helix (α_A_; residues 120–131), and formation of helix in α_B_ is necessary for the affinity maintaining, although the helix formation of α_A_ is not [25]. The NMR study [27] has shown that the orientation of α_A_ relative to the KIX framework is disordered, although that of α_B_ is well determined with contacting tightly with KIX. Contrarily, in the unbound state, α_A_ has a higher helix propensity than α_B_ [30]. These features should be confirmed through simulations.

As described in this paper, we performed TTP-McMD simulations of pKID in the presence and absence of KIX. We denote the residues 120–131 as “α_A_ residues” and the residues 134–145 as “α_B_ residues” whether these residues form helices in simulation snapshots or not. Similarly, when the α_A_ and α_B_ residues are expressed as elements, they are denoted respectively as an “α_A_ region” and “α_B_ region.” We show that the obtained conformational ensemble from the simulation agrees with the experimental features described above, and that the α_A_ and α_B_ regions have different mechanisms of coupled folding and binding. 

## 2. Experimental Section

### 2.1. Setting Simulation Systems (pKID and pKID–KIX Systems)

We designated the simulation system of pKID in the absence of KIX as the “pKID system” and that in the presence of KIX as the “pKID–KIX system.” In the NMR experiment on the pKID–KIX complex [27], the pKID sequence was longer than that deposited in PDB (residues 119–146) because unstructured regions are not deposited. We used the deposited pKID region for the simulation, which is the minimum sequence of pKID binding with KIX (residues 586–666).

We prepared the pKID system as follows. The coordinates of pKID were taken from the first model out of the 17 NMR ones. The pKID was immersed in a solvent sphere (called sphere 1; radius = 30 Å), which consists of water molecules with the density of 1 g/mL equilibrated at 300 K in advance. The mass center of pKID was set to the center of sphere 1, and water molecules overlapping with pKID were removed. To neutralize the net charge of the system and make consistency with the ionic strength of the NMR experiment, nine water molecules were selected randomly and replaced with five Cl^−^ and four Na^+^ ions. Finally, the pKID system consisted of 3473 water molecules, pKID, and nine ions. Although pKID was taken from the NMR model at this stage, the structure was randomized completely using a high-temperature simulation, as described later.

Next, we prepared the pKID–KIX system as follows. The coordinates of the two proteins were taken from the first NMR model again. They were immersed in a solvent sphere (called sphere 2; radius = 37 Å). The sphere 2 radius was sufficiently large to contain the entire KIX structure, as described later. The center of sphere 2 was set to the mass-center position of pKID of the NMR model. It is noteworthy that the center of sphere 2 is fixed in space (*i.e.*, the center of sphere 2 is not fixed to pKID) when pKID is moving in the simulation: The translation and rotation of pKID are not restrained. Water molecules overlapping the proteins were removed. Eighteen randomly selected water molecules were replaced with nine Cl^−^ and nine Na^+^ ions. Then, the pKID–KIX system came to consist of 6166 water molecules, pKID, KIX, and 18 ions. This complex was dissociated by a high-temperature simulation, as shown later.

**Figure 1:**
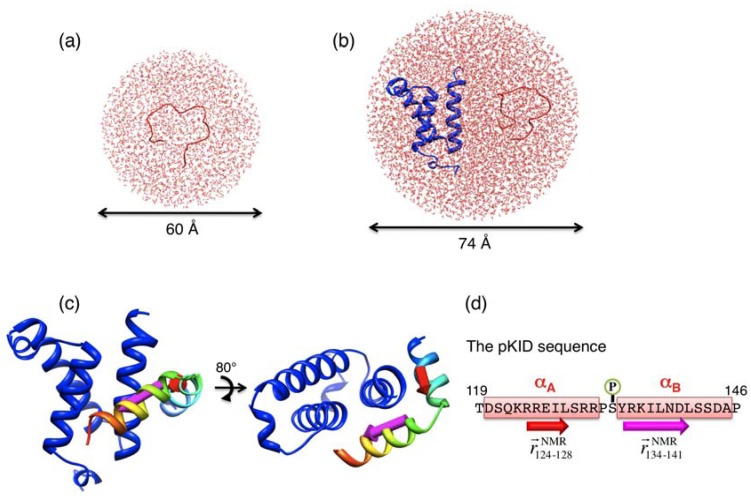
Initial structures for TTP-McMD simulations of the pKID system (**a**) and the pKID–KIX system (**b**). Blue and red ribbons respectively represent KIX and pKID. Small molecules surrounding the proteins are solvent molecules. Arrows below the spheres indicate the sizes of solvent spheres. (**c**) First NMR model, where KIX is represented by blue ribbon and pKID by rainbow (blue N-terminal; red C-terminal). Red and magenta arrows indicate vectors 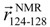 and 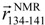, respectively, which are defined in Section 3.2. (**d**) pKID sequence, where α_A_ and α_B_ residues are highlighted and character P in the circle represents the phosphate group. Arrows below the sequence indicate the starting and ending residues to define the vectors 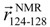 and 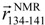. The structure images were created using Chimera viewer software [39].

We used the AMBER-based hybrid force field for the proteins. This force field is the combination of AMBER force field parm94 (*E*_94_) [31] and parm96 (*E*_96_) [32] with a mixing rate *ω* as *E* = (1-*ω*)×*E*_94 _+*ω*×*E*_96_ . We set *ω*= 0.75 because our previous works indicated that *ω*= 0.75 reproduces the optimal secondary structure preference for some peptides [33,34]. The TIP3P model [35] was used for the water molecules. After energy minimization, we performed a high-temperature (700 K) canonical MD simulation for each of the pKID and pKID–KIX systems to generate the initial simulation conformations for the following TTP-McMD. Figure 1 shows that this temperature was sufficiently high to demolish the native conformation of pKID and to dissociate the native complex. Although pKID was able to move freely in the solvent spheres, the structure of KIX was weakly restrained in the simulation of the pKID–KIX system, as described later. We used the simulation program PRESTO ver. 3 [36]. The canonical MD and TTP-McMD simulations were done in the following condition: 1.0 fs time step, SHAKE algorithm [37] to constrain covalent bonds between heavy atoms and hydrogen atoms, and the cell-multipole expansion method [38] to compute the long-range electrostatic interactions. Throughout the simulation, we maintained the volume of sphere 1 and 2 by supplementing a harmonic potential on atoms flying out of the sphere to pull them into the sphere. Additionally, for the pKID–KIX system, the positions of the main-chain atoms (N, Cα, C, and O) of KIX residues (591–594, 597, 608–611, 617–621, 623–640, 646–648, 660–661) were restrained on those of the NMR model by a weak harmonic potential to maintain the KIX structure around the NMR model. No restraint was applied on pKID to diffuse it freely in sphere 2. Figure 1(a) and Figure 1(b) are the last snapshots of the high-temperature canonical MD simulations of the pKID and pKID-KIX systems, respectively, that are used for the initial structures in TTP-McMD simulations.

### 2.2. Trivial Trajectory Parallelization of Multicanonical Molecular Dynamics (TTP-McMD)

Before introducing TTP-McMD, we describe the conventional McMD, *i.e.*, the single-run McMD. The single-run McMD is a canonical simulation at a temperature *T* (a constant-temperature method [40] was used as thermostat in this study) using the multicanonical energy *E*_mc_ instead of the original potential energy *E* of the system, as


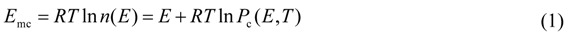


Where *n*(E) is the density of states of the system, *R* signifies the gas constant, *P*_c_(*E*,*T*) the probability distribution function of the canonical ensemble at *T*. If the simulation is sufficiently long, then the single-run McMD provides a flat distribution along the axis of *E* because the probability distribution is formally given by the following equation.


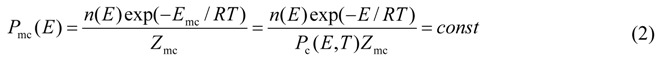


In that equation, *Z*_mc_ is the partition function for McMD, which can be regarded simply as a factor to normalize the distribution function *P*_mc_ To derive Equation (2), we used a formulation of statistical mechanics: *P*_c_(*E*,*T*)=*n*(*E*)exp(-*E*/RT)/Z_c_, where *Z*_c_ is a factor to normalize *P*_c_(*E*,*T*) *i.e.*, the partition function of the system). This flat energy distribution guarantees that the conformation can overcome the energy barriers and visit low energy conformational regions during the simulation. We refer to the entire conformational ensemble obtained from McMD as a “multicanonical ensemble.” Then we can reconstruct a canonical energy distribution *P*_c _at any target temperature *T*_tag_ from *P*_mc_ as


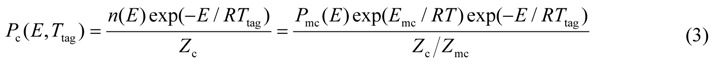


To derive Equation (3), we used Equation (2) in the following form: *n*(*E*)=*Z*_mc_*P*_mc_(*E*)exp(*E*_mc_/*RT*). The canonical conformational ensemble at *T*_tag_ is constructed by assigning the probability *P*_mc_(*E*,*T*_tag_) to all conformations in the multicanonical ensemble.

McMD uses the probability density function *P*_c_(*E*,*T*) in Equation (1), which is generally unknown *a priori* (before simulation). Then, we must construct it self-consistently through iterative simulation runs, during which *P*_c_(*E*,*T*) converges to a precise function. TTP-McMD takes an advantage over the single-run McMD [19,20]. TTP-McMD is technically equivalent with performing independent multiple runs of single-run McMD starting from various initial conformations. The multiple trajectories generated are integrated simply into an ensemble. It is noteworthy that low-energy conformations (low-energy basins) distribute widely in the conformational space, which are spaced by high-energy barriers. Then, a single-run McMD takes a long flight while overcoming the barriers to reach the low-energy basins. On the other hand, the multicanonical algorithm tries to ensure the flat distribution along the energy axis (Equation (2)) so that the conformation is expected to exist evenly in both the low-energy and high-energy regions. This evenness might cause a difficulty of single-run McMD, *i.e.*, no convergence. The connected multiple runs (TTP-McMD), which are spread in the conformational space, are equivalent to a long trajectory, each part of which flights and searches the low-energy basins. Consequently, TTP-McMD provides the convergence of conformational ensemble faster than the single-run McMD.

The initial conformations for TTP-McMD were those shown in Figure 1(a) and (b), which were generated from the high-temperature canonical MD simulations, as described above. In this study, we performed 256 multiple runs for each system. To obtain the accurate *P*_c_(*E*,*T*) in Equation (1), we performed the simulations iteratively. First, we performed a canonical simulation at 700 K and generated the probability *P*_c_(*E*,700K) for each system. This probability distribution is restricted around the peak position (designated as *E*_high_) of *P*_c_(*E*,700K), and is accurate only in the narrow region. We designate this energy range as [*E*_0_,*E*_high_] (*E*_0_<*E*_high_), where *E*_0 _is a lower limit of the accurately sampled energy region. Second, we performed the first TTP-McMD at 700 K with the multicanonical energy *E*_mc_, where *P*_c_(*E*,700K) obtained above was used for *P*_c_(*E*,*T*) in Equation (1). In the simulation, we set artificial energy walls at *E*_0 _and *E*_high_ so that the conformation did not escape out of the range [*E*_0_,*E*_high_]. This simulation produced a flat energy distribution *P*_mc_(*E*) only in [*E*_0_,*E*_high_]. Then, using Equation (3) we reconstructed the probability *P*_c_(*E*,200K). Here, *E*_mc_ defined by *E* + *RT* ln *P*_c_(*E*,200K) is effective only for multicanonical runs at 200 K. The reason for this temperature reset (*i.e.*, 700 K → 200 K) is explained later. We again extrapolated *P*_c_(*E*,200K) to an energy range as [*E*_1_,*E*_high_] (*E*_1_<*E*_0_), and performed multicanonical runs at 200 K to produce a flat energy distribution *P*_mc_(*E*) in this extrapolated energy range. Multicanonical runs were performed iteratively until the sampled energy range reaches an energy (*E*_low_) that corresponds to a temperature lower than a room temperature. After reaching *E*_low_, we performed the final TTP-McMD to generate a flat energy distribution in [*E*_low_,*E*_high_]. We used 256 computing cores (intel Xeon X5365 3.0 GHz), each core executed one run of TTP-McMD. In the equilibration stage (*i.e.*, the stage to estimate *E*_mc_ before the final sampling stage), the simulations were done for 21 ns and 23 ns in each of 256 trajectories (total of 5.4 μs and 5.9 μs) of the pKID and pKID-KIX system, respectively. The final runs were done for 8.51 ns in each of 256 trajectories (total time of 2.18 μs) and stored the snapshots every 5 ps for subsequent conformational analyses. The computation times for the equilibration stage were 33 and 76 days for the pKID and pKID-KIX system, respectively. Those for the final sampling stage were 13 and 28 days for the pKID and pKID-KIX system, respectively.

As described above, we reset the simulation temperature from 700 K to 200 K. The multicanonical energies at the two temperatures are, respectively, *E*_mc_(700K)=700×*R* ln *n*(*E*) and *E*_mc_(200K)=200×*R* ln *n*(*E*). Mathematically, the multicanonical ensembles from *E*_mc_(700K) and *E*_mc_(200K) are expected to be equivalent:


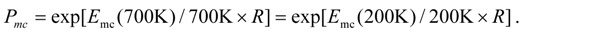
.

Consequently, the temperature reset is theoretically non-sense. However, the two simulations produce practically different ensembles. We use a polynomial function to approximate *P*_c_(*E*,*T*) in Equation (1). The function form *P_c_*(*E*,200*K*) is smoother than *P_c_*(*E*,700*K*). Then,* P_c_*(*E*,200*K*) is more suitable than *P_c_*(*E*,700*K*) to define *E*_mc_.

## 3. Results and Discussion

The conformational ensembles at 315 K of the pKID and pKID–KIX systems were investigated. In Section 3.1, we analyzed the pKID system and showed that pKID is intrinsically disordered in the unbound state. In Section 3.2, we investigated the pKID–KIX system and showed that the free energy landscape of pKID in the bound state is rugged. Furthermore, the orientation of α_A_ of pKID relative to the KIX framework fluctuates more than α_B_ does. In Section 3.3, we discuss the mechanism of the coupled folding and binding for this system. In the final section, differences between the unbound and bound states are discussed.

### 3.1. The Conformational Ensemble in the pKID System (Unbound State)

**Figure 2:**
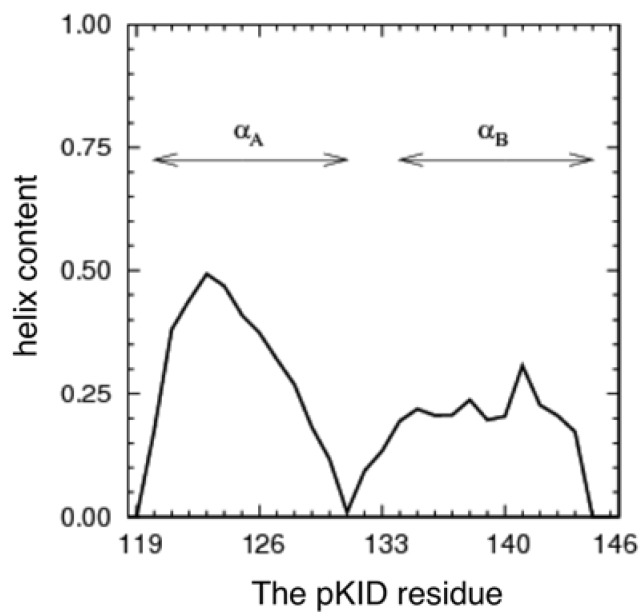
α-helix content rate per residue in the pKID system computed from conformations in the 315K-ensemble. DSSP program [41] was used to assign the α-helix to each residue. The x-axis represents the residue ordinal number of pKID in the original PDB file [27]. Arrows indicate the α_A_ and α_B_ regions.

The TTP-McMD simulation for the pKID system is explored evenly in an energy range [−35000.0 kcal/mol, −23480.0 kcal/mol] that corresponds to a temperature range [300 K, 700 K]. From the multicanonical ensemble, we constructed a conformational ensemble at 315 K (denoted as “315K-ensemble”) consisting of 9922 conformations. It is noteworthy that 315 K is the temperature of the NMR experiment [27]. The average of energy *E* at 315 K was −34272.0 kcal/mol. The secondary structure content at each residue position is shown in Figure 2. Apparently, the α_A_ region preferred helix more than the α_B_ region does. This result agrees with the NMR observation [30]. However, the helix content rate was small: The content for α_A_ was below 50% and that for α_B_ was about 20%, and the unbound pKID was fluctuating among the helix and non-helix conformations at room temperature. Furthermore, the relative positioning between the α_A_ and α_B_ regions fluctuated highly in the 315K-ensemble. Consequently, the simulation confirmed that pKID in the unbound state is intrinsically disordered.

**Figure 3:**
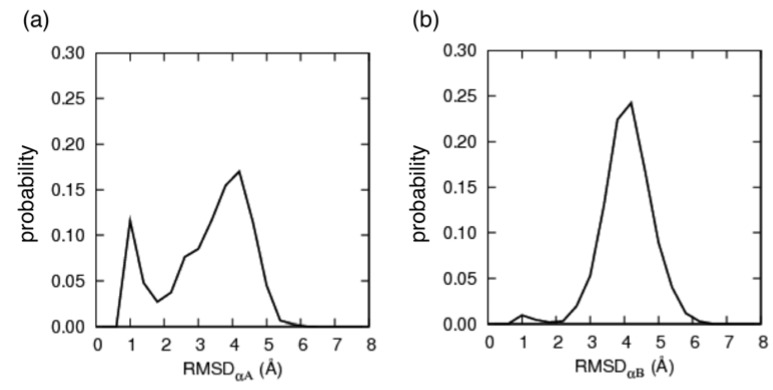
Probability distributions of (**a**) *RMSD*_αA_ and (**b**) *RMSD*_αB_ in the pKID system at 315 K.

We investigated whether the conformation in the unbound state showed similarity with that in the native bound state (*i.e.*, NMR structure). Picking up a sampled conformation from the 315 K-ensemble, the root-mean-square deviation (*RMSD*) was calculated for each of the α_A_ and α_B_ regions as follows: After superimposing Cα atoms of the α_A_ region onto those in each of the 17 NMR models, 17 *RMSD* values were calculated and the smallest *RMSD* of the 17 values, denoted as *RMSD*_αA_, was selected. Similarly, the smallest *RMSD*, denoted as *RMSD*_αB_, was calculated for the α_B_ region. The Cα-atomic *RMSD* for the entire pKID (residues 121–144) is denoted as *RMSD*_all_, where raw coordinates of pKID were used for the RMSD computation without superposition. Figure 3 presents the probability distributions of *RMSD*_αA_ and *RMSD*_ΑB_ for all conformations in the 315K-ensemble. A non-negligible peak was found in the α_A_ region at small *RMSD*_αA_:* RMSD_α__A_* ≈ 1.0 Å. Contrarily, the α_B_ region showed a small peak at *RMSD*_αB_≈ 1.0 Å. These results suggest that the α_A_ and α_B_ regions have different mechanisms of coupled folding and binding: α_A_ might bind with KIX with a population-selection mechanism, where the structured fraction of α_A_ is used for binding to KIX. In contrast, α_B_ might bind to KIX with an induced-folding mechanism, where α_B_ is bindable to KIX with various conformations and then the native complex is formed.

### 3.2. Free Energy Landscape and Orientation of α_A_ in the pKID–KIX System

**Figure 4:**
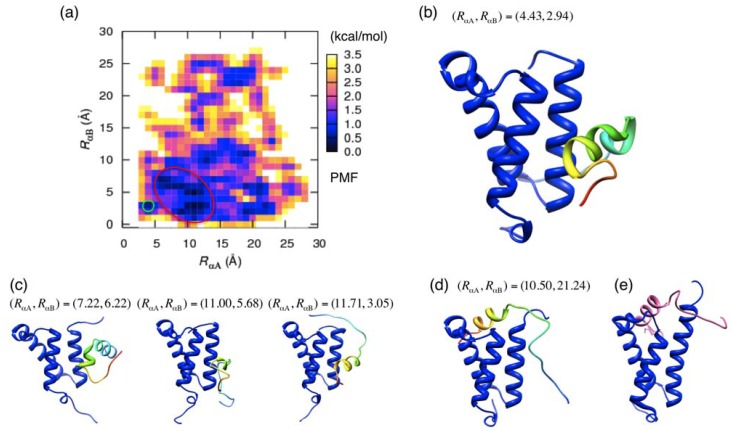
(**a**) Free energy landscape (potential of mean force (*PMF*) defined by Equation (4)) constructed on the plane of *R*_αA_ and *R*_αB_. The lowest *PMF* was set to zero. The green and red circles are described in the text. (**b**) Smallest-*RMSD*_all_ (nearest-native) structure of pKID in the pKID–KIX system. KIX is represented by the blue ribbon, and pKID by rainbow (blue N-terminal and red C-terminal). Values for *R*_αA_ and *R*_αB_ shown near the structures indicate its position in panel **a**. (**c**) Structures taken from the red-circle region of panel **a**. (**d**) pKID structure bound at the MLL binding site of KIX via the α_B_ region. (**e**) MLL (magenta) structure bound to KIX (blue) taken from PDB ID: 2agh.

The TTP-McMD simulation of the pKID–KIX system produced a flat energy distribution in an energy range [−64440.0 kcal/mol, −42500.0 kcal/mol], which corresponds to a temperature range [300 K, 700 K]. From the multicanonical ensemble, we derived the 315K-ensemble (8840 conformations). The free energy landscape for the α_A_ and α_B_ regions on binding to KIX was constructed as a function of two variables *R*_αA_ and *R*_αB_ defined as follows: 
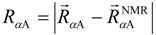
 and 
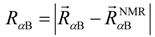
, where 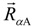 and 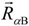 respectively represent the position vectors of the centroids of the α_A_ and α_B_ regions in a sampled conformation, and 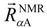 and 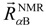 those in the reference structure (*i.e.*, the NMR model), respectively. Using these distances as the reaction coordinates, we calculated the potential of mean force (PMF) as


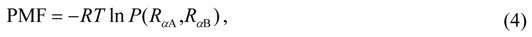


where the probability *P*(*R*_αA_,*R*_αB_) was represented as the number of conformations counted in a fraction [*i* ± 0.5 Å, *j* ± 0.5 Å] (*i*,*j * = 0,1,2,∙∙∙) of [*R*_αA_,*R*_αB_] over the total conformations (8840) in the 315-K ensemble, *R* is the gas constant, and *T* is the temperature (315 K). Figure 4(a) shows the free energy landscape *PMF*, which was complex and ragged. We found a low free-energy fraction circled by green circle around (*R*_αA_,*R*_αB_)=(4 Å, 3 Å). We designate this fraction as the “native-like low-free-energy fraction.” This native-like fraction comprised conformations with *RMSD*_all_<7.1 Å. The nearest-native structure, shown in Figure 4(b), had *RMSD*_all_ of 5.65 Å, whose position in Figure 4(a) is: (*R*_αA_,*R*_αB_)= (4.43 Å, 2.94 Å). This structure forms a partially disordered α_A_ helix and a well-ordered α_B_ helix.

We also found a non-native broad fraction with low free energy in Figure 4(a) (red circle): [*R*_αA_,*R*_αB_]= [5–13 Å, 0–7 Å]. We designate this fraction “non-native low-free-energy fraction”, where pKID attached KIX with various binding poses as exemplified in Figure 4(c). Because the two fractions were distinguishable in Figure 4(a), a free energy barrier exists between them. The non-native low-free-energy fraction suggests that pKID can bind to KIX with a structural diversity, and the generated various encounters reach the final native-like fraction across the free energy barrier. This result suggests that the high flexibility of pKID might help the pKID–KIX association because pKID binds to KIX without adopting a well-ordered structure. The final native-complex formation is completed after forming the various non-native complexes. Later in this report, we describe our examination of why the non-native low-free-energy fraction was larger than the native-like low-free-energy fraction in the free-energy landscape and why the α_A_ helix is partially disordered, even in the native-like low-free-energy fraction (Figure 4(b)).

We found pKID bound to another site of KIX in 323 snapshots of the 315K-ensemble (Figure 4(d)). This site is a binding site for the activation domain of the mixed lineage leukemia (MLL) transcription factor [42]. The MLL–KIX complex structure (Figure 4(e)) shows that a segment of MLL adopts helix and binds to KIX, and the other parts are unstructured. In all of these snapshots, the α_B_ region adopted helix to bind to the MLL binding site with the α_A_ region unstructured. The orientation of the α_B_ helix cylinder was approximately the same as that of the MLL segment in the MLL–KIX complex structure [43]. Consequently, the α_B_ region corresponds to the MLL segment. In fact, both the MLL-binding site and the genuine α_B_-binding site on the KIX surface consist of hydrophobic amino acids. Furthermore, the hydrophobic residues in the α_B_ region and the MLL segment have similarity; they contains *ϕ*-x-x-*ϕ*-*ϕ* motif (*ϕ* = hydrophobic residue and x = any residue), which is conserved in many KIX binding proteins (see Figure 9 of reference [43]). It is particularly interesting that in the presence of MLL, pKID binds to KIX with the two-fold higher affinity than pKID in the absence of MLL [44]. Our simulation results suggest that MLL might facilitate the pKID binding to the genuine binding site via blocking the MLL binding site.

The orientations of the α_A_ and α_B_ regions with respect to the KIX framework were investigated, respectively, using inner products, *I*_αA_ and *I*_αB_. The inner product *I*_αA_ was defined as


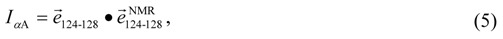


where vectors 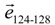 and 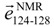 respectively represent the unit vectors of vectors 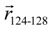 and 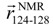: see Figure 1(c). The vector 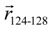 is pointing from the Cα-atomic position of the 124th residue to that of the 128th residue in a sampled conformation. The vector 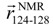 is defined in the same way for the NMR structure. For the orientation of α_B_ residues, *I*_αB_ was defined as 


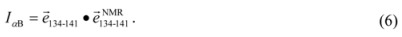


The unit vectors 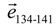 and 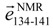 were calculated similarly as 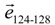 and 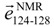 by replacing the residue numbers 124–134 and 128–141. When the inner products *I*_αA_ and *I*_αB_ are 1, the orientations are aligned as in the native bound form.

**Figure 5:**
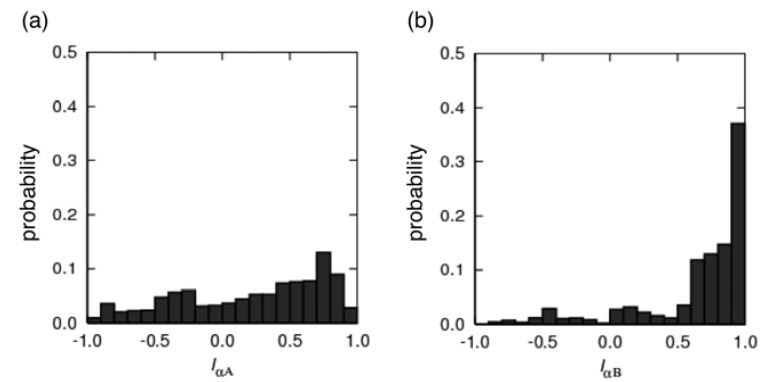
Orientational probability distribution of (**a**) α_A_ and (**b**) α_B_ regions. Definitions for *I*_αA_ and *I*_αB_ are given in the text (Equations 5 and 6).

The inner products were calculated for 2476 conformations whose distances [*R*_αA_,*R*_αB_] satisfy *R*_αA_≤13 Å and *R*_αB_≤7 Å, which involves the native-like (green circle in Figure 4(a)) and non-native (red circle) low-free-energy fractions. The histogram for *I*_αB_ (Figure 5(b)) has the largest peak at 1, which indicates that the α_B_ region attaches to KIX with the same orientation as that in the final bound form in both low-free-energy fractions. We discussed the factors stabilizing this orientation later in Section 3.4. Recall that the α_B_ region less adopts helix than the α_A_ region in the isolated state (Figure 2) and that the α_B_ region seldom adopts native-complex form in the isolated state (Figure 3(b)). Consequently, it is likely that the α_B_ region binds to KIX with the right orientation and then the helix is formed. In contrast, the α_A_ region has a large variety in the orientation (Figure 5(a)). These results agree well with the experimental observation: The α_B_ orientation ordered well and the α_A_ orientation does less in the native complex form [27]. Furthermore, the simulation results are consistent with the experimental report that the α_B_ region has a stronger binding affinity than the α_A_ region to bind to KIX [25].

### 3.3. Correlation between Intermolecular Residue Contacts and Folding of pKID

The folding of α_A_ and α_B_ regions upon binding was investigated by measuring residue contacts between pKID and KIX. An intermolecular contact was determined as the distance between the centers of side chains of two residues: If this distance is below 6.5 Å, then we judge that the two residues are contacting. We calculated the contacts (“native contacts”) in the 17 NMR models, where a contact is assigned to a residue pair if the pair is contacting in at least 8 of the 17 models. We found 30 native contacts: 8 between α_A_ and KIX residues, 18 between α_B_ and KIX residues, and 4 between the other residues in pKID and KIX residues. We designate contacts other than the native contacts found in simulation snapshots as “non-native contacts”.

**Figure 6:**
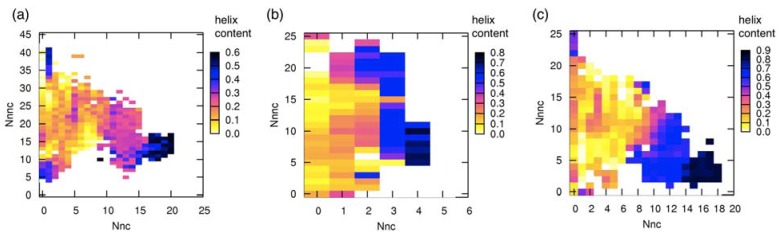
Helix content of pKID at 315 K projected on plane of *N*nc and *N*nnc. Results are shown for the entire pKID (**a**), for the α_A_ region (**b**), and for the α_B_ region (**c**).

We counted the quantities of the native and non-native contacts, respectively denoted as *N*nc and *N*nnc, for each conformation. Figure 6 presents the helix content rate in pKID at 315 K projected on the *N*nc-*N*nnc plane. Coupled folding and binding for α_B_ is explained well from Figure 6(c): In complexes where the native contacts are less formed, various non-native contacts are formed. With increasing native contacts, the non-native contacts decrease. Finally the full native contacts are formed with a few (*ca*. 5) non-native contacts. This result suggests that coupled folding and binding of the α_B_ region accords to the induced-fit mechanism, where the α_B_ region varies the conformation in the encounter complex to reach the native complex. Because the helix content rate of α_B_ is small in the unbound state (Figure 3(b)), the induced-fit mechanism has an advantage over the population-selection mechanism. In fact, the formation of the non-native contacts before the native contacts might facilitate the pKID–KIX association, as discussed earlier in the Introduction: the α_B_ region can bind to KIX via non-native contacts without waiting until a helix is formed.

However, the α_A_ folding depends less on *N*nc than the α_B_ folding did. A few native contacts (*ca.* 4, which is 50% of the entire native contacts) were able to stabilize the binding of α_A_ to KIX. This result derives from the high directional fluctuations of α_A_, as shown in Figure 5(a). This result agrees qualitatively with those of the NMR experiment [27], where the flexibility of α_A_ is greater than α_B_. One can recognize that α_A_ has directional diversity by viewing the NMR models (PDB ID: 1kdx). Furthermore, the fragile contacts between α_A_ and KIX qualitatively support and agree with the experiment [25] showing that the binding affinity assigned to α_A_ is weaker than that to α_B_. We were unable to specify which of the induced fit or population selection characterizes the α_A_ binding better, although Figure 3(a) supports the population selection. The large flexibility of α_A_ prevents us from answering which is likely. However, we emphasize that the dynamics [27] and the weak binding affinity [25] of α_A_ are characterized by its large flexibility.

### 3.4. Change of Accessible Surface Area on Binding

**Figure 7:**
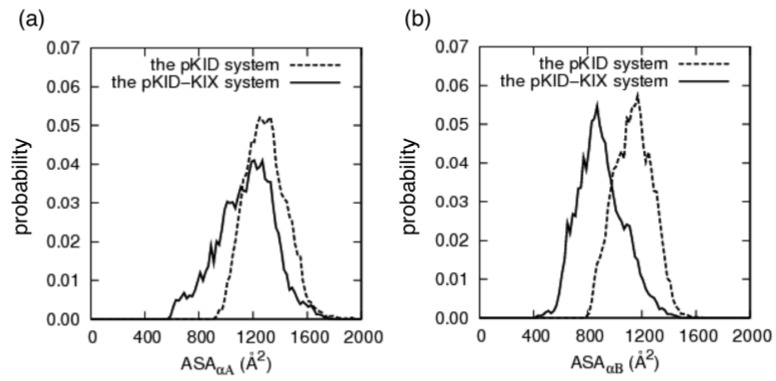
Normalized probabilities of the accessible surface area (**a**) for α_A_ (*ASA*_αA_) and (**b**) for α_B_ residues (*ASA*_αB_). Dashed and solid lines respectively represent the probabilities for the pKID and pKID–KIX systems.

We demonstrated the change of accessible surface area (ASA) on binding. We denote ASAs for the α_A_ and α_B_ regions respectively as *ASA*_αA_ and *ASA*_αB_. The distributions of *ASA*_αA_ and *ASA*_αB_ are presented in Figure 7. The average of *ASA*_αB_ was 1136 Å^2^ for the pKID system and 889 Å^2^ for the pKID–KIX system. Consequently, the reduction of ASA upon binding is clear for α_B_ (Figure 7(b)). This reduction was caused mainly by hydrophobic contacts formed in the complex. However, for α_A_ the reduction was small (Figure 7(a)): the average *ASA*_αA_ was 1294 Å^2^ in the pKID system and 1150 Å^2^ in the pKID–KIX system. This small reduction occurs because the α_A_ region has a large helix probability in the pKID system (Figure 2) and the α_A_-KIX interaction is weak (Figure 5(a)). These results are consistent with experimental observations [25], showing that α_A_ and α_B_ regions are characterized, respectively, by weak and strong binding affinities.

The averages of *ASA*_αA_ and *ASA*_αB_ over the 17 NMR models (complex form) are, respectively, 1093±72 Å^2^ and 723±52 Å^2^. Consequently, the computed ASA is larger than the experimental value because the computed 315K-ensemble consisted of various complex conformations, as shown in the non-native low-free-energy fraction (Figure 2(c)) and even the conformations in the native-like low-free-energy fraction partially disordered as in Figure 4(b).

**Figure 8:**
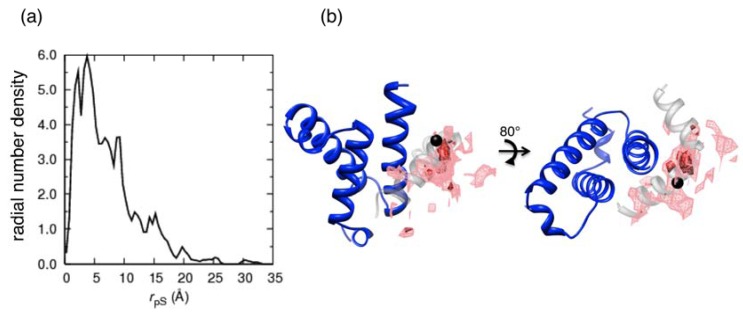
Distribution of the Cα-atom of the phosphorylated SER133 (pS133) in the pKID–KIX system at 315 K. (**a**) Radial (*r*_pS_) distribution function defined as: 
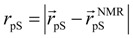
 where 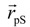 denotes the Cα-atom position of pS133 in the sampled snapshots and 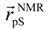 one in the reference structure (NMR model 1). (**b**) Spatial distribution of the Cα-atomic position of pS133, where red and magenta contours respectively represent the iso-density regions with density larger than 0.0032 Å^–3^ and 0.0001 Å^–3^. The black sphere is the position in the NMR Model 1.

A hydrophobic interaction is a non-directional interaction. However, the α_B_ residues were able to bind KIX with the same orientation as in the native complex form (Figure 5(b)). This directional conservation is inferred from a spatial distribution of the phosphorylated SER (pS133), which is located in the middle of the α_A_ and α_B_ (Figure 1(d)). The pS133 interacted with a hydrophilic residue (Y658) and a positively charged residue (K662) of KIX. Figure 8(a) depicts that pS133 restrained the hydrophobic interaction of the α_B_ helix with KIX as in the native complex form. Figure 8(b) demonstrates the spatial distribution of the Cα-atomic position of pS133, where the iso-density contours were computed for conformations with *R*_αA_ ≤ 13 Å and *R*_αB_ ≤ 7 Å. Although the high-density site was slightly deviated from that in the NMR structure, the anchor effect of pS133 is clearly shown. Consequently, phosphorylation plays an important role for specifying the α_B_-helix orientation (Figure 5(b)). We presume that this phosphorylation-induced restraint on the α_B_ helix increases the binding affinity more than non-phosphorylated KID [25,26].

## 4. Conclusions

We performed the TTP-McMD simulations for the pKID folding and binding with KIX in explicit solvents. Although the overall property of pKID in the unbound state was disordered, pKID has the nascent helix propensity in the α_A_ and α_B_ regions in the computed conformational ensemble. The propensity for α_A_ was stronger than that for α_B_, which agrees with an experiment described in the literature [30].

In the presence of KIX, the free-energy landscape at 315 K involved two low-free-energy fractions: The native-like low-free-energy fraction and non-native low-free-energy fraction. Because the α_B_ region can bind to KIX with various non-native contacts (various encounter complexes), the α_B_ region might provide fast association with KIX [8]. This landscape proposes an induced-fit mechanism for coupled folding and binding of the α_B_ region: various encounter complexes are possible in the early stage, and the complex passes through the free-energy barrier to reach the native-like low-free-energy fraction. The well-oriented binding of the α_B_ region was controlled by the phosphorylated SER133 located in the middle of the α_A_ and α_B_ regions. This control supports the higher binding affinity of pKID than KID, as observed experimentally [25,26]. In contrast to the α_B_ region, the α_A_ region exhibited high flexibility, which agrees qualitatively with that found in the NMR structure [27]. An earlier experiment [25] has demonstrated that the helix formation of α_A_ is not important for binding to KIX. Consequently, the simulation supports the high binding affinity of α_B_ and the low binding affinity of α_A_.

It is particularly interesting that the α_B_ region bound to another shallow hydrophobic concave of KIX than the genuine pKID-binding site, where α_B_ adopted helix. This hydrophobic concavity is the MLL binding site of KID, to which a segment of MLL binds with adopting helix [42]. It has been pointed out in an earlier report that the hydrophobic residue pattern of the α_B_ and MLL segments have a similar hydrophobic amino-acid residue pattern [43]. In the presence of MLL, pKID binds KIX with the two-fold higher affinity than pKID alone [44]. We presume that MLL might facilitate the pKID binding to the genuine binding site by blocking the MLL binding site.

The current study demonstrated the importance of hydrophobic interactions between pKID and KIX. Because the multicanonical simulation is an efficient sampling method, the multicanonical trajectory can overcome high potential energy barriers in the conformational space. At the cost of this high performance, the trajectory does not provide information of time series. A conventional MD simulation may provide another important aspect, such as electrostatic interaction, on the complex formation. 

Finally, it is noteworthy that the non-native low-free-energy fraction (red circle in Figure 4(a)) is larger than the native-like low-free-energy fraction (green circle) in the free-energy landscape and that the α_A_ helix is partially disordered, even in the native-like low-free-energy fraction (Figure 4(b)). These points disagree with the NMR experimentally obtained results [27]. Presumably, this disagreement results from the truncation of pKID, which are unstructured in the NMR experiment. It is important to remember that the computed pKID segment is the only part deposited to PDB. The unstructured region might stabilize the α_A_ helix more, which might result in an increase of the native-like low-free-energy fraction.
